# Alterations in the jejunal microbiota and fecal metabolite profiles of rabbits infected with *Eimeria intestinalis*

**DOI:** 10.1186/s13071-022-05340-5

**Published:** 2022-06-26

**Authors:** Xu Yuan, Jin Liu, Xiaofen Hu, Shanshan Yang, Shengwei Zhong, Tingyu Yang, Yunxiao Zhou, Guotong Zhao, Yijie Jiang, Yong Li

**Affiliations:** 1 College of Animal Science and Technology, Jiangxi Agricultural University, Nanchang, 330045 Jiangxi China; 2 Dezhou Agricultural and Rural Bureau, Dezhou, 253000 Shandong China

**Keywords:** *E. intestinalis*, Jejunum, Histopathology, Gut microbiota, Metabolome

## Abstract

**Background:**

Rabbit coccidiosis is a major disease caused by various *Eimeria* species and causes enormous economic losses to the rabbit industry. Coccidia infection has a wide impact on the gut microbiota and intestinal biochemical equilibrium. In the present study, we established a model of *Eimeria intestinalis* infection in rabbits to evaluate the jejunal microbiota and fecal metabolite profiles.

**Methods:**

Rabbits in the infected group were orally inoculated with 3 × 10^3^ *E. intestinalis* oocysts. On the eighth day of infection, jejunal contents and feces were collected for 16S rRNA gene sequencing and liquid chromatography–tandem mass spectrometry (LC–MS/MS) analysis, respectively. Jejunum tissues were harvested for hematoxylin and eosin (H&E), periodic acid-Schiff (PAS), and immunohistochemistry (IHC) staining.

**Results:**

Histopathological analysis showed that the whole jejunum was parasitized by *E. intestinalis* in a range of life cycle stages, and PAS staining showed that *E. intestinalis* infection caused extensive loss of goblet cells. IHC staining revealed that TNF-α expression was higher in the *E. intestinalis* infection group. Moreover, both the jejunal microbiota and metabolites significantly altered after *E. intestinalis* infection. At the genus level, the abundances of *Escherichia* and *Enterococcus* significantly increased in the infected group compared with the control group, while those of *Oscillospira*, *Ruminococcus*, *Bacteroides*, *Akkermansia*, *Coprococcus*, and *Sarcina* significantly decreased. In addition, 20 metabolites and two metabolic pathways were altered after *E. intestinalis* infection, and the major disrupted metabolic pathway was lipid metabolism.

**Conclusions:**

*Eimeria intestinalis* infection induced intestinal inflammation and destroyed the intestinal homeostasis at the parasitized sites, leading to significant changes in the gut microbiota and subsequent corresponding changes in metabolites.

**Graphical Abstract:**

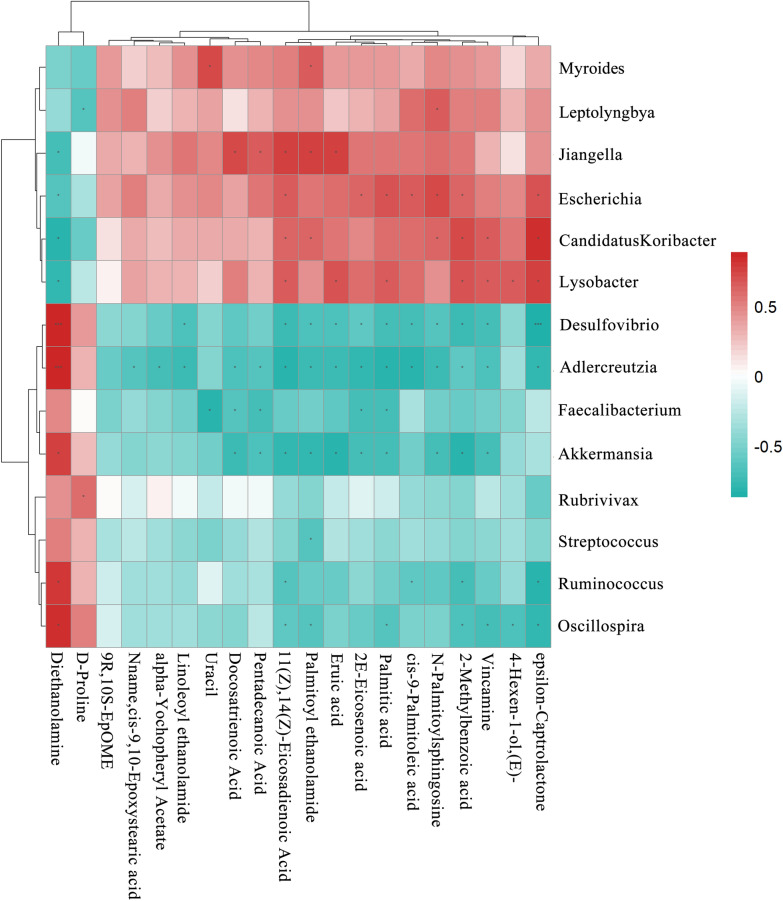

## Background

Rabbit coccidiosis is a common disease caused by a variety of *Eimeria* species parasitizing the liver and intestinal epithelial cells. It is harmful to rabbits at different ages and seriously affects the development of the rabbit industry [1]. Among the 15 *Eimeria* species that infect rabbits [2], *Eimeria intestinalis* is considered the most pathogenic [3]. It can cause anorexia, weight loss, diarrhea, and even death in infected rabbits [2, 4]. *Eimeria intestinalis* infection destroys intestinal epithelial cells and triggers an inflammatory response in the gut. In addition, it induces a T helper 1 (Th1) immune response and increases the levels of pro-inflammatory and anti-inflammatory cytokines such as tumor necrosis factor alpha (TNF-α), interferon gamma (IFN-γ), interleukin 12 (IL-12), IL-6, and IL-10 [5–7].

The gut microbiota plays a very important role in metabolic, nutritional, physiological, and immunological processes [8, 9]. Moreover, symbiotic bacteria can modulate intestinal immune responses, prevent inflammatory diseases [10], reduce the adhesion of intestinal pathogens, and stimulate the proliferation of the gut epithelium [11]. *Eimeria* invasion damages intestinal epithelial cells and disrupts intestinal homeostasis [12]. *Eimeria* infection was found to increase intestinal colonization of other bacterial pathogens, such as Enterobacteriaceae [13], Bacteroidales, and *Rikenella* [14]. Although significant damage to rabbit intestines caused by *E. intestinalis* has been reported, the influence of *E. intestinalis* infection on the gut microbiota and metabolites needs further study.

It is well established that gut microbes and their metabolic products regulate host metabolism. As feces are the final product of intestinal digestion, the fecal metabolome provides valuable information for the discovery of potential biomarkers related to intestinal disorders [15]. Metabolomics, which can comprehensively describe the host’s metabolic responses to infections by parasites, has been widely applied in parasitological studies. For example, Ma [16] identified 33 metabolites strongly correlated with *Toxoplasma gondii* infection in the hippocampus, and 30 of these were deemed potential biomarkers for infection. Altered fatty acid metabolism and β-oxidation were identified as dominant metabolic signatures associated with *Eimeria acervulina* infection [17]. In addition, blood-borne *Candida albicans* caused the depletion of several probiotic bacteria and increased the number of pathogenic bacteria. Pearson correlation analysis revealed that some altered intestinal bacteria were highly related to changes in fecal metabolites [18].

In this study, changes in jejunal histopathology and the jejunal microbiota and metabolites were comprehensively explored in rabbits infected with *E. intestinalis* by combining 16S ribosomal RNA (rRNA) gene sequencing and fecal metabolomic analyses. *Eimeria intestinalis* infection damaged the intestinal barrier, disrupted the gut microbiota at the parasitized site, and altered the related metabolites. The alterations in the gut microbiota and metabolites play an important role in the diagnosis of coccidiosis and help us to better understand the pathogenesis of coccidiosis.

## Methods

### Rabbits and oocysts

Twenty 30-day-old weaned rabbits were purchased from Ganzhou Animal Husbandry Research Institute and raised under coccidia-free conditions in sterilized wire cages. They were randomly divided into two groups of 10 rabbits per group: the infected group and the control (non-infected) group. *Eimeria intestinalis* oocysts (Guangxi strain) were donated by the College of Veterinary Medicine, South China Agricultural University. After propagation, collection, and sporulation, oocysts were preserved in potassium dichromate. Before infection, oocysts were washed with normal saline solution and centrifuged 3 times to remove the potassium dichromate. The feces of these rabbits were examined daily to confirm the absence of any parasitic infection. At 45 days of age, the rabbits in the infected group were orally inoculated with 3 × 10^3^ *E. intestinalis* oocysts [4], while the rabbits in the control group were inoculated with the same volume of PBS.

Six rabbits from each group were euthanized for the collection of jejunal contents and fecal samples on the eighth day after infection; the samples were quickly frozen in liquid nitrogen after retrieval and were then stored at −80 °C until further analysis. Jejunum tissues were immediately harvested and fixed with 4% formaldehyde.

### Hematoxylin–eosin (H&E) and periodic acid-Schiff (PAS) staining

Jejunum tissues were subjected to dehydration through a graded ethanol series, cleared with xylene, embedded in paraffin, and sliced into 5-μm-thick sections. The integrity of the jejunal microstructure was analyzed by H&E staining. In addition, PAS staining was used to detect mucus-containing goblet cells, and images were acquired under a light microscope.

### Immunohistochemistry (IHC) staining

The steps for IHC staining were previously described [19] and are briefly listed below. Sections were incubated with 3% H_2_O_2_ for 10 min to block endogenous peroxidase activity and were then incubated with normal goat serum as a blocking reagent for 30 min. The sections were then incubated overnight at 4 °C with a TNF-α antibody (dilution 1:600, Proteintech Group, Inc.). Later, the sections were incubated with horseradish peroxidase-conjugated secondary antibodies (anti-mouse immunoglobulin G [IgG] polymer) for 30 min prior to DAB staining and hematoxylin counterstaining. Phosphate-buffered saline (PBS) solution (0.01 mol/l) was used instead of the primary antibody as a control group, and the other steps were the same as described above. The DAB staining intensity was analyzed using Image-Pro Plus (version 6.0) and represented by the mean optical density (MOD) value (MOD = IOD/area, IOD: integrated optical density).

### Genomic DNA extraction and 16S rRNA gene sequencing

Total genomic DNA from the samples was extracted using the cetrimonium bromide (CTAB) method. The DNA concentration and purity were evaluated on 1% agarose gels. Specific barcoded primers and high-fidelity DNA polymerase were used for polymerase chain reaction (PCR) amplification. The V3-V4 region of the bacterial 16S rRNA gene was amplified using the primers 341F (5′-ACGACGGAGGGCATCCTCA-3′) and 806R (5′-GGACTACHVGGGTWTCTAAT-3′). All PCRs were carried out in a 30 µl reaction volume containing 15 µl of Phusion^®^ High-Fidelity PCR Master Mix (New England Biolabs). The thermal cycling parameters for PCR amplification were set as follows: initial denaturation at 98 °C for 1 min; 30 cycles of denaturation at 98 °C for 10 s, annealing at 50 °C for 30 s, and extension at 72 °C for 60 s; and a final extension step at 72 °C for 5 min. After detection by 2% gel electrophoresis, PCR products were mixed at an equal density ratio, and the mixture was then purified with an AxyPrep DNA Gel Extraction Kit. Sequencing libraries were generated using an NEBNext^®^ Ultra™ DNA Library Prep Kit for Illumina (NEB, USA) according to the manufacturer’s recommendations, and index codes were added. Finally, the libraries were sequenced on the Illumina Miseq and HiSeq 2500 platforms.

Paired-end reads generated from the original DNA fragments were merged using FLASH. QIIME (V1.7.0) software was used to analyze the sequences and filter the raw data. Chimeric sequences were removed using the UPARSE-OTU and UPARSE-OTUref algorithms, and sequences with ≥ 97% similarity were assigned to the same operational taxonomic units (OTUs). In-house Perl scripts were used to analyze alpha and beta diversity. Unweighted UniFrac distance for principal coordinate analysis (PCoA) and non-metric multidimensional scaling (NMDS) were calculated by QIIME to visually evaluate the overall difference and similarity of bacterial communities. To confirm the differences in the abundances of individual taxonomic units between the infected group and control group, linear discriminant analysis effect size (LEfSe) analysis was used to measure biomarkers within different groups, and LEfSe [linear discriminant analysis effect size] LDA > 2 and *P* < 0.05 were considered to indicate statistical significance.

### Fecal metabolome analysis

After the samples were thawed slowly at 4 °C, an appropriate amount of each sample was added to a precooled methanol/acetonitrile/water (2:2:1, v/v) solution. The mixture was centrifuged for 15 min (14,000×*g*, 4 °C), and the supernatant was dried in a vacuum centrifuge. For LC–MS analysis, samples were redissolved in 100 μl of solvent (acetonitrile/water = 1:1, v/v), vortexed, and centrifuged for 15 min (14,000×*g*, 4 °C). Quality control samples were prepared by pooling 10 µl of each sample and analyzed together with the other samples. For hydrophilic interaction liquid chromatography (HILIC) separation, samples were analyzed using a 2.1 mm × 100 mm ACQUITY UPLC BEH 1.7 µm column (Waters, Ireland). For both electrospray ionization (ESI)-positive and ESI-negative modes, the mobile phase contained solvent A (25 mM ammonium acetate and 25 mM ammonium hydroxide in water) and solvent B (acetonitrile). The flow rate was 0.5 ml/min, and the elution gradient was as follows: 1 min, 85% B; 11 min, 65% B; 4 min, 40% B; 5 min, 85% B.

The raw MS data (wiff.scan files) were converted to mzXML format using ProteoWizard MSConvert and were then imported into publicly available XCMS software. After normalization to the total peak intensity, the processed data were analyzed with an R package (ropls) by orthogonal partial least-squares discriminant analysis (OPLS-DA) and partial least-squares discriminant analysis (PLS-DA). The robustness of the model was evaluated by permutation test. The variable importance in the projection (VIP) value of each variable in the OPLS-DA model was calculated to indicate its contribution to the classification. Student’s *t*-test was further used to determine the significance of each metabolite with a VIP value > 1, and *P* < 0.05 was considered to indicate statistical significance. Pathway analyses were carried out based on the Kyoto Encyclopedia of Genes and Genomes (KEGG, http://www.kegg.jp/) Pathway Database.

### Correlation analysis of gut microbiota and metabolites

Spearman correlation analysis was used to calculate the correlation coefficients between significantly different microbes and metabolites selected from experimental samples. Matrix heatmap, hierarchical clustering, correlation network, and other analyses were performed with the Pearson algorithm in R (version 3.5.1) and with Cytoscape (version 3.5.1) to explore the interactions between the microbiota and the metabolites from multiple perspectives.

## Statistical analysis

Student’s *t*-test in SPSS software (version 25) was used for statistical analysis. The values are expressed as the means ± standard errors (SEs), and *P* < 0.05 was considered significant.

## Results

### Histopathological changes in the jejunum of rabbits infected with *E. intestinalis*

**Fig. 1:**
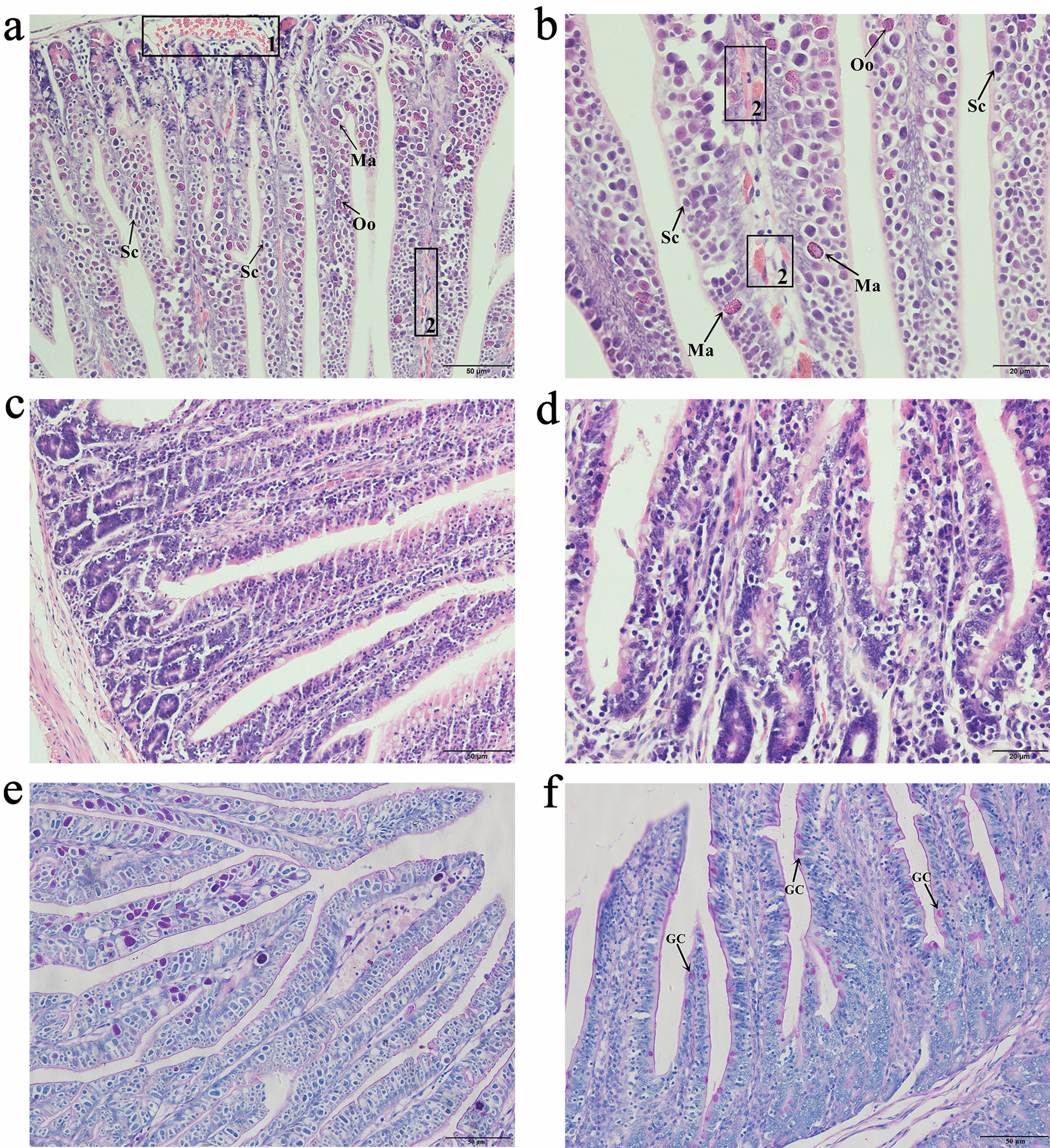
Pathological changes in the rabbit jejunum on the eighth day after *E. intestinalis* infection. **a**, **b** *E. intestinalis* infection group at 200 × and 400 × magnification, respectively. **c**, **d** Control group at 200 × and 400 × magnification, respectively. **e**, **f** The goblet cells in the jejunum of the *E. intestinalis* infection group and control group at 200 × magnification, respectively. Sc, schizont; Ma, macrogametocyte; Oo, oocyst; GC, goblet cell. Rectangle 1 indicates congestion, rectangles 2 indicate hemorrhage

The jejunal structure of rabbits in the control group was intact, with a clear outline of intestinal epithelial cells, but jejunal histopathological analysis showed severe changes in the *E. intestinalis* infection group. The jejunum tissues, from the intestinal villi to the glands, were occupied by a large number of oocysts and other life cycle stages of *E. intestinalis*, among which schizonts were the most abundant and could be easily seen at 400 × magnification. The villi were completely destroyed, and many discrete hemorrhagic lesions were visible in the lamina propria. The submucosal blood vessels showed severe congestion (Fig. 1a–d). The number of goblet cells in the jejunum decreased compared with that in the control group (Fig. 1e, f).

### TNF-α expression in the jejunum of rabbits infected with *E. intestinalis*

**Fig. 2:**
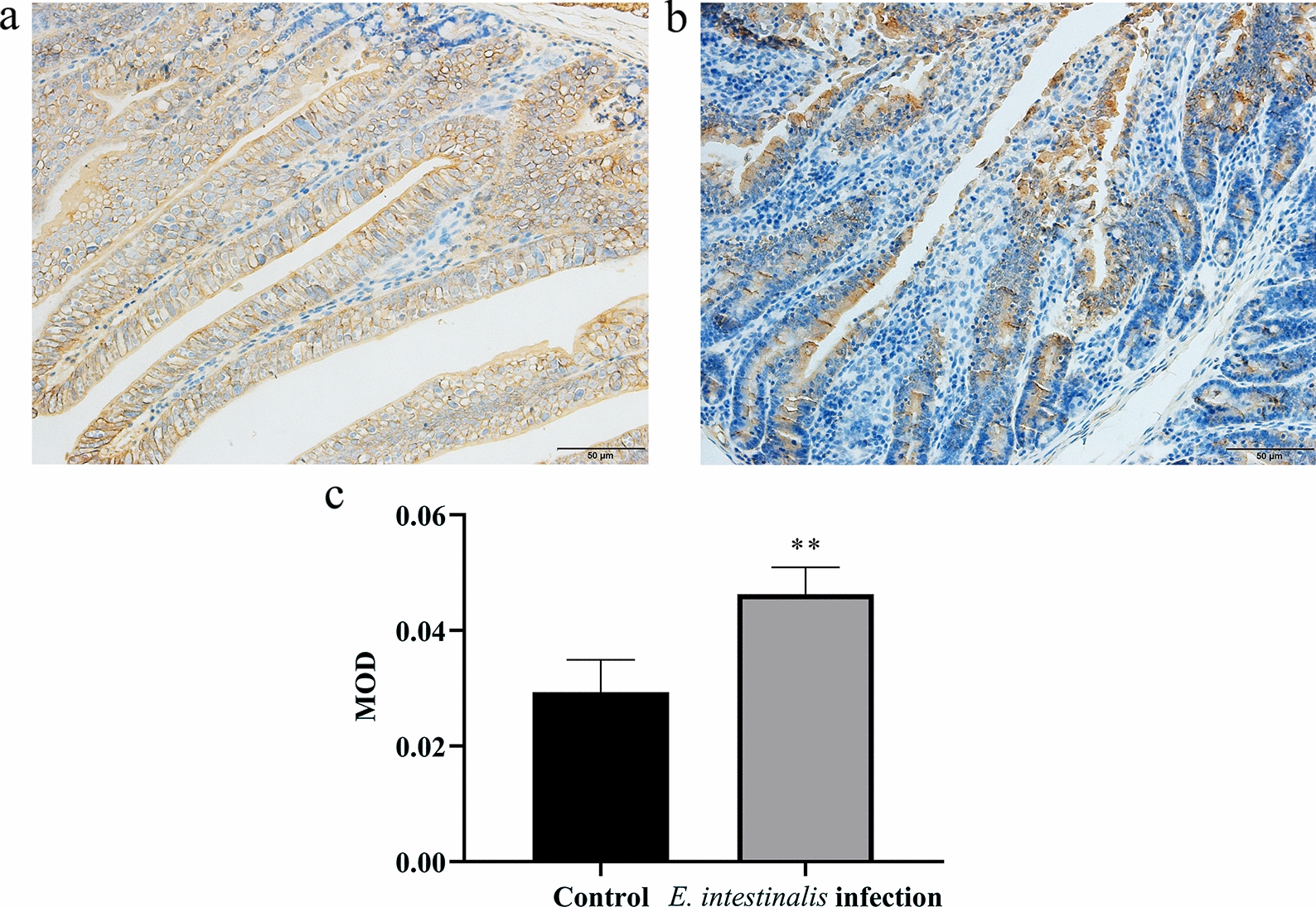
TNF-α expression in the jejunum on the eighth day after *E. intestinalis* infection. **a** *E. intestinalis* infection group. **b** Control group. **c** TNF-α protein expression levels; *P < 0.05, **P < 0.01

TNF-α in the jejunum was mainly distributed in the intercellular contents within the villi and submucosa, appearing yellow to brown in color, with punctate or granular staining (Fig. 2a, b). TNF-α protein expression significantly increased in *E. intestinalis*-infected rabbits compared with control rabbits (*P* < 0.05) (Fig. 2c).

### The microbiota composition in the jejunum of rabbits infected with *E. intestinalis*

**Fig. 3:**
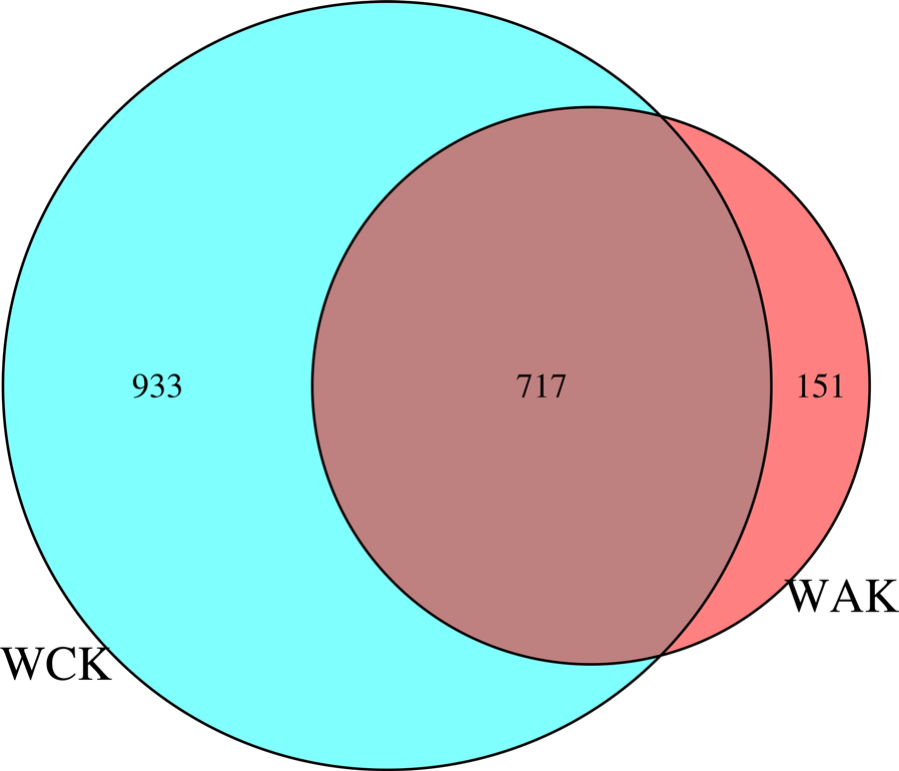
Venn diagram based on the OTU distribution. WAK, *E. intestinalis* infection group; WCK, control group

**Fig. 4:**
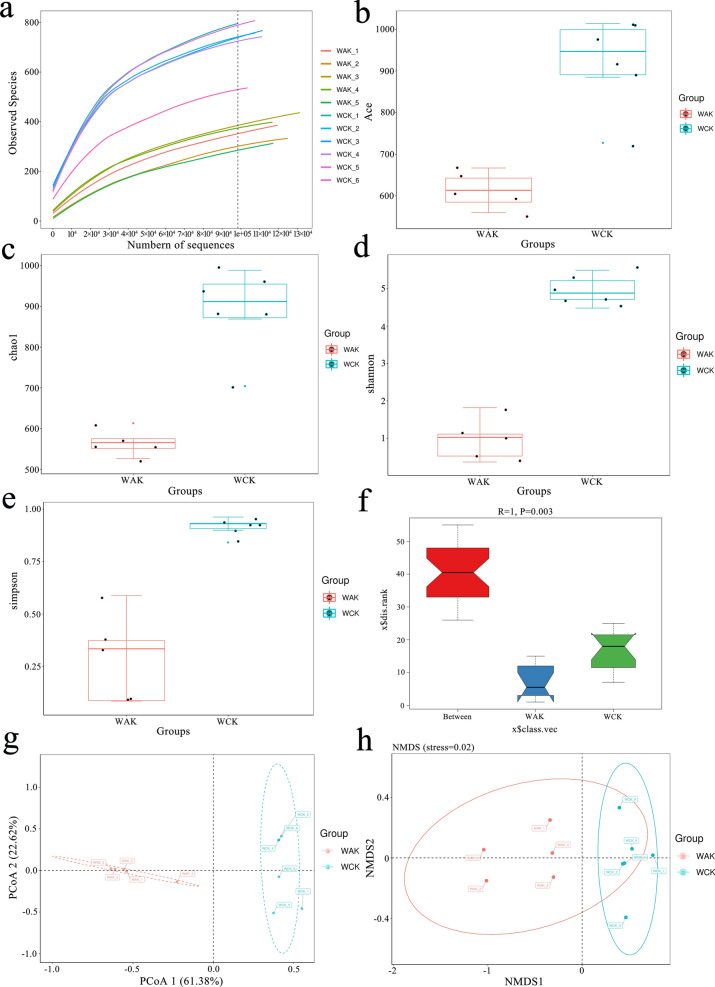
Diversity analysis of the jejunal microbiota. **a** Rarefaction curve reflecting the abundances of species in the samples. **b** ACE index and **c** Chao1 index as estimates of OTU richness. **d** Shannon diversity index and **e** Simpson index as estimates of species diversity. **f** ANOSIM analysis based on the Bray–Curtis dissimilarity distance matrix. **g** PCoA based on the weighted UniFrac distance and **h** NMDS of the Bray–Curtis analysis indicating a clear separation between the two groups

A total of 1801 OTUs were identified from all samples on the basis of 97% nucleotide sequence similarity, and 717 OTUs were shared by both groups (Fig. 3). Rarefaction curves suggested that the sequencing depth was near saturation and reflected the rationality of the sequencing data (Fig. 4a). The ACE and Chao1 indices indicated that the species abundance was reduced in the *E. intestinalis* infection group compared with the control group (Fig. 4b, c). In addition, the higher Shannon and Simpson indices indicated higher bacterial diversity, which meant that the microbial diversity decreased in the *E. intestinalis* infection group compared with the control group (Fig. 4d, e). Furthermore, analysis of similarities (ANOSIM) showed that the differences between the two groups were greater than those within the groups (*R*-value = 1, *P* = 0.003) (Fig. 4f). Together, these results clearly indicate that the diversity of the jejunal microbiota was reduced in the *E. intestinalis* infection group. The jejunal microbiota composition of the two groups was obviously separated, as shown by principal coordinate analysis (PCoA) based on the weighted UniFrac distance and non-metric multidimensional scaling (NMDS) (Fig. 4g, h).

**Fig. 5:**
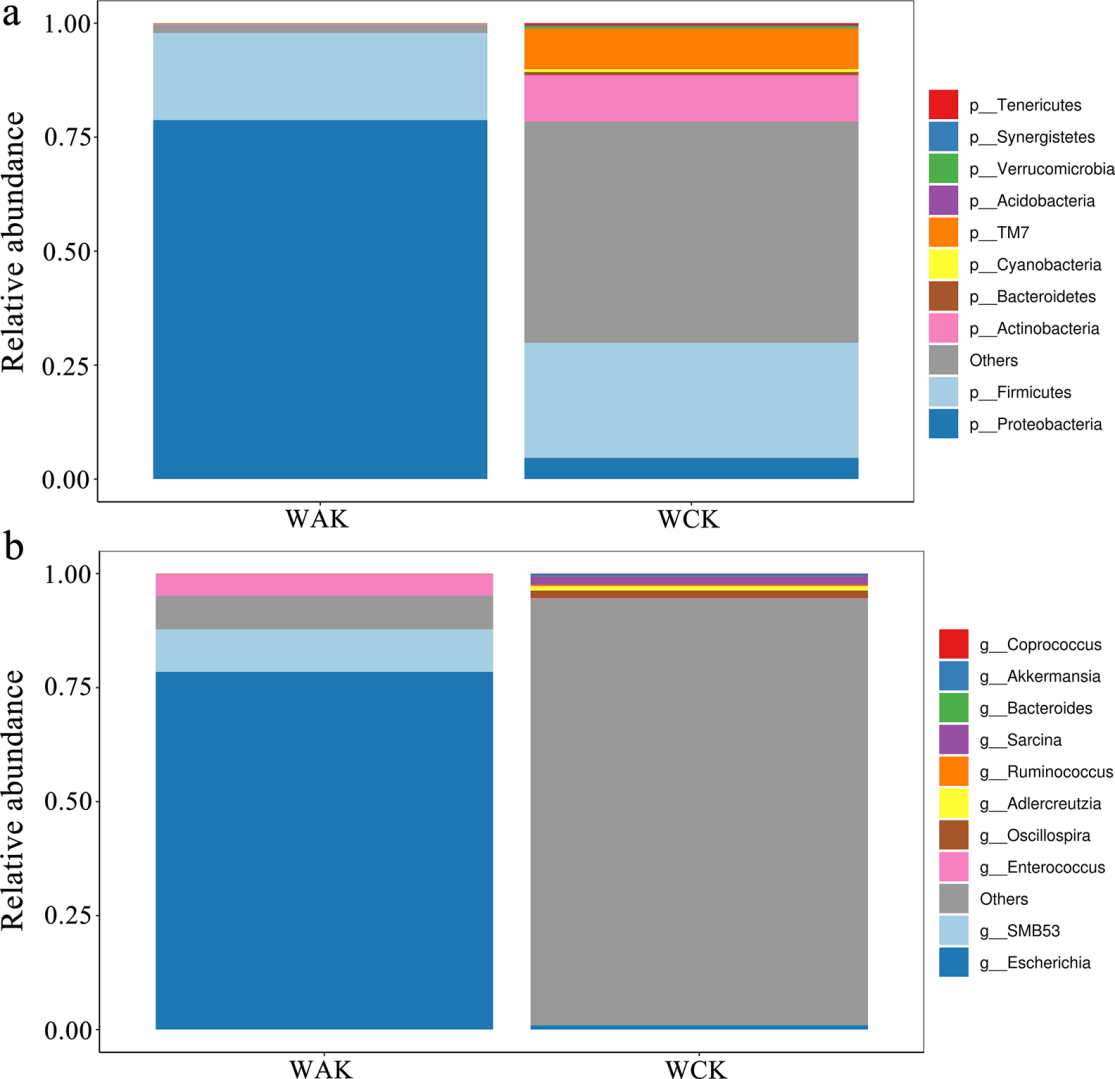
Jejunal microbiota alterations at the phylum and genus levels. **a** Phylum level. **b** Genus level. The histograms show the top 10 bacterial phyla and genera in terms of relative abundance, and “Others” indicates bacteria with abundance not among the top 10 abundances by classification

**Fig. 6:**
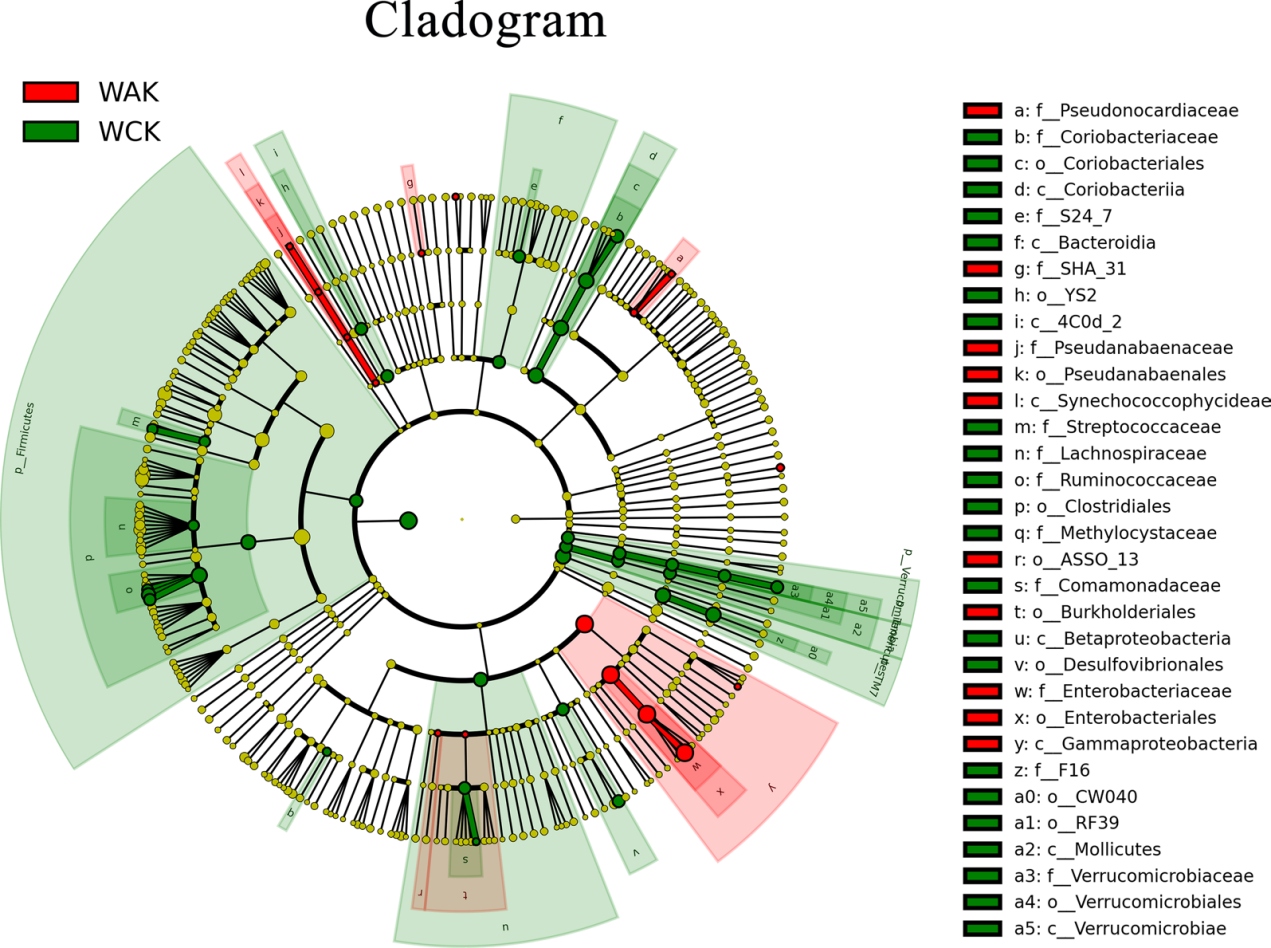
Cladogram of differentially abundant bacterial taxa in the gut microbiota. The radiating circles from inside to outside represent the classification levels from phylum to genus (or species)

**Fig. 7:**
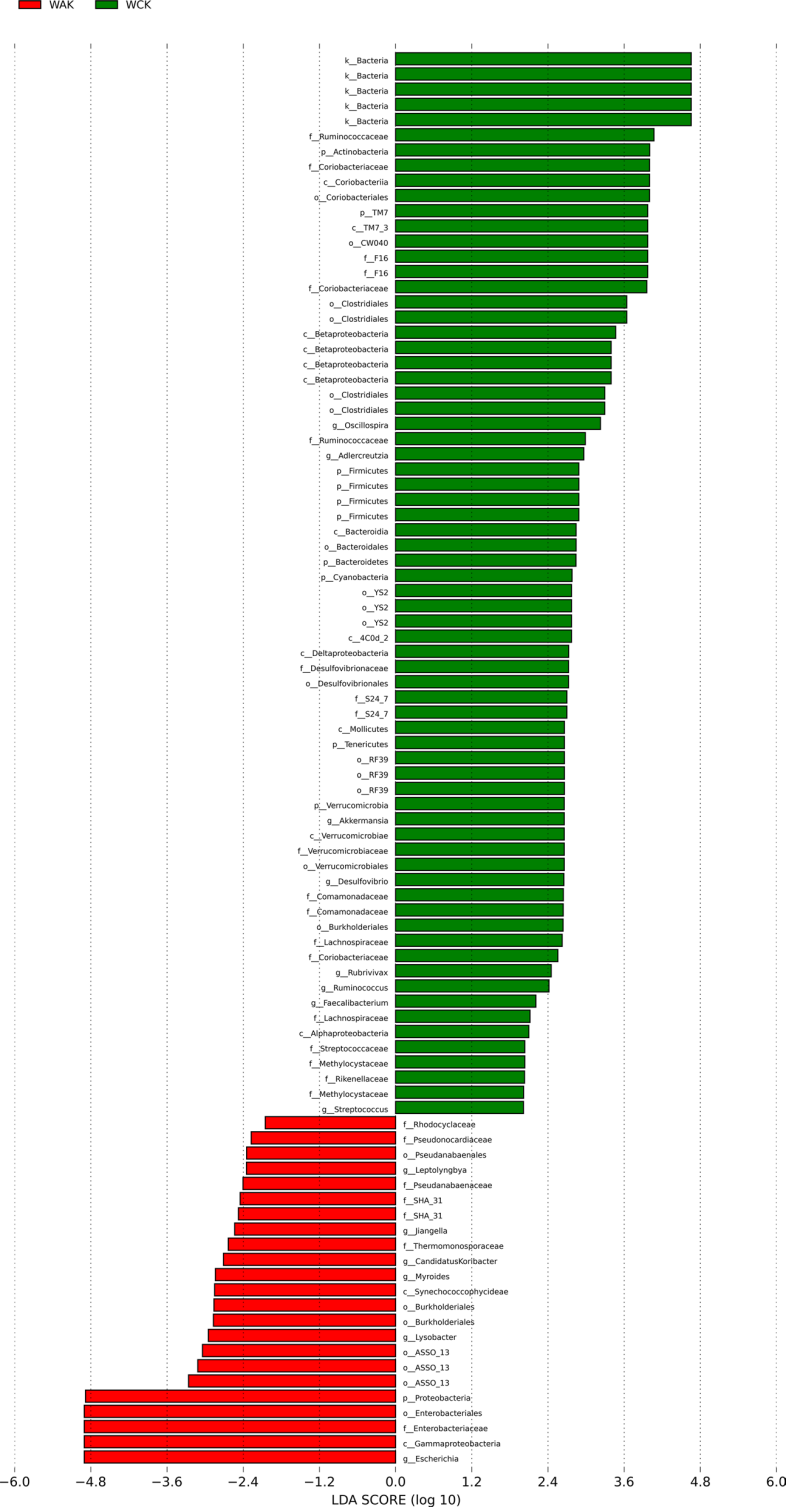
Histogram of LDA scores. The taxa with LDA scores > 2 are shown

To evaluate the composition of the gut microbiota, we compared the relative abundances at the phylum and genus levels. At the phylum level, Firmicutes (25.3%) was the most predominant phylum in the control group, followed by Actinobacteria (10.2%) and TM7 (9.0%). A clear increase in the Proteobacteria (78.8%) abundance was found in the *E. intestinalis* infection group. In contrast, the relative abundance of Firmicutes decreased from 25.3% in the control group to 19.1% in the *E. intestinalis* infection group (Fig. 5a). Among the 10 most dominant genera, *Escherichia* (1.0% vs 78.5%), *Enterococcus* (0.1% vs 4.7%), and *SMB53* (9.3%) were significantly more abundant in the infected group than in the control group (*P* < 0.05), while *Oscillospira*, *Ruminococcus*, *Bacteroides*, *Akkermansia*, *Coprococcus*, and *Sarcina* were significantly less abundant (Fig. 5b). A cladogram for different classification-level abundances based on the LEfSe result is shown in Fig. 6. In total, 14 genera with LDA scores > 2 were identified (Fig. 7).

### The fecal metabolite profiles in rabbits infected with *E. intestinalis*

**Fig. 8:**
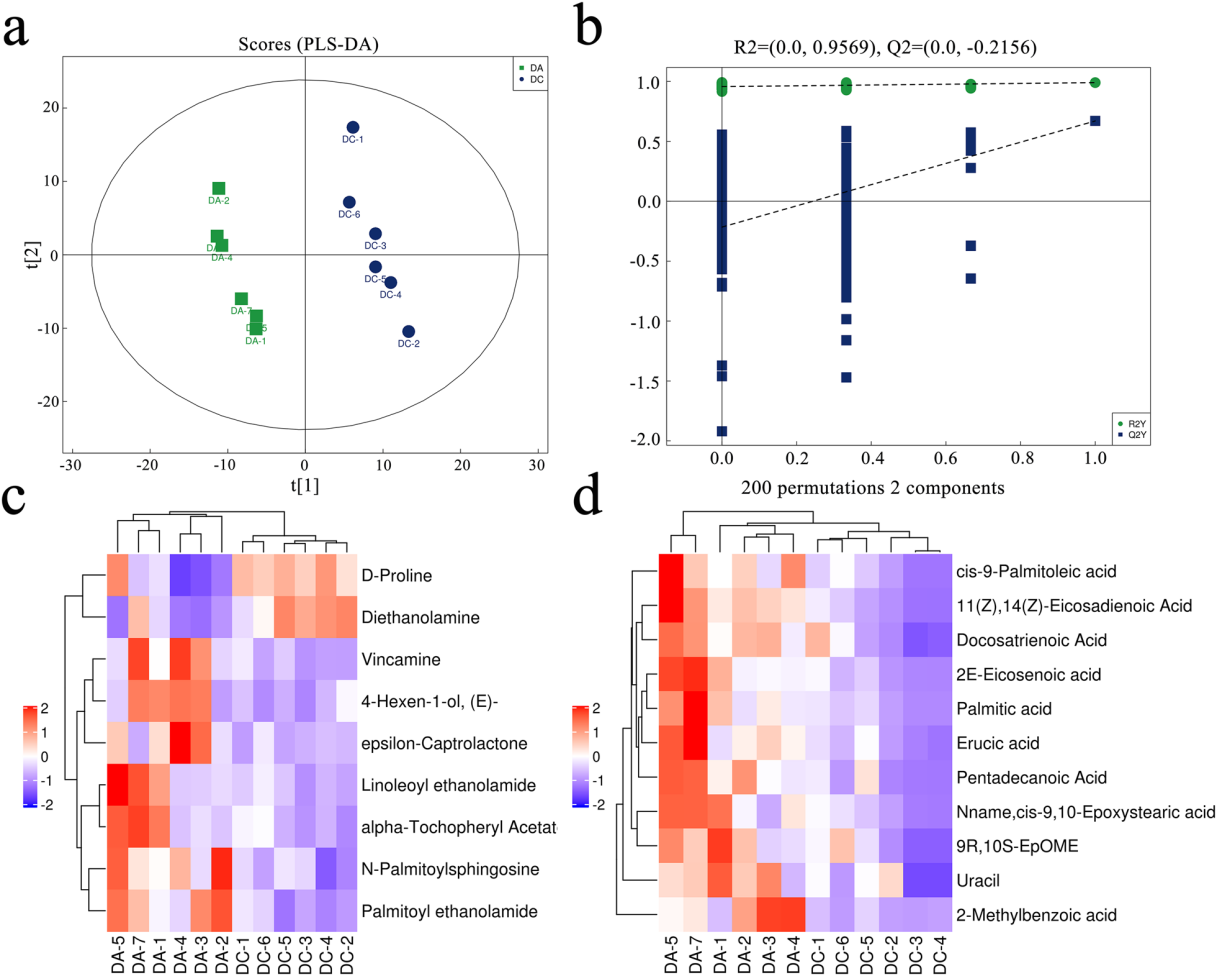
Multivariate analysis of metabolites. **a** PLS-DA score plots of fecal samples from the control and *E. intestinalis* infection groups in ESI+ mode. **b** Permutation test of PLS-DA model in ESI+ mode. **c**, **d** Hierarchical clustering heatmaps of significantly different metabolites in ESI+ and ESI- modes, respectively. DA, *E. intestinalis* infection group; DC, control group

**Fig. 9:**
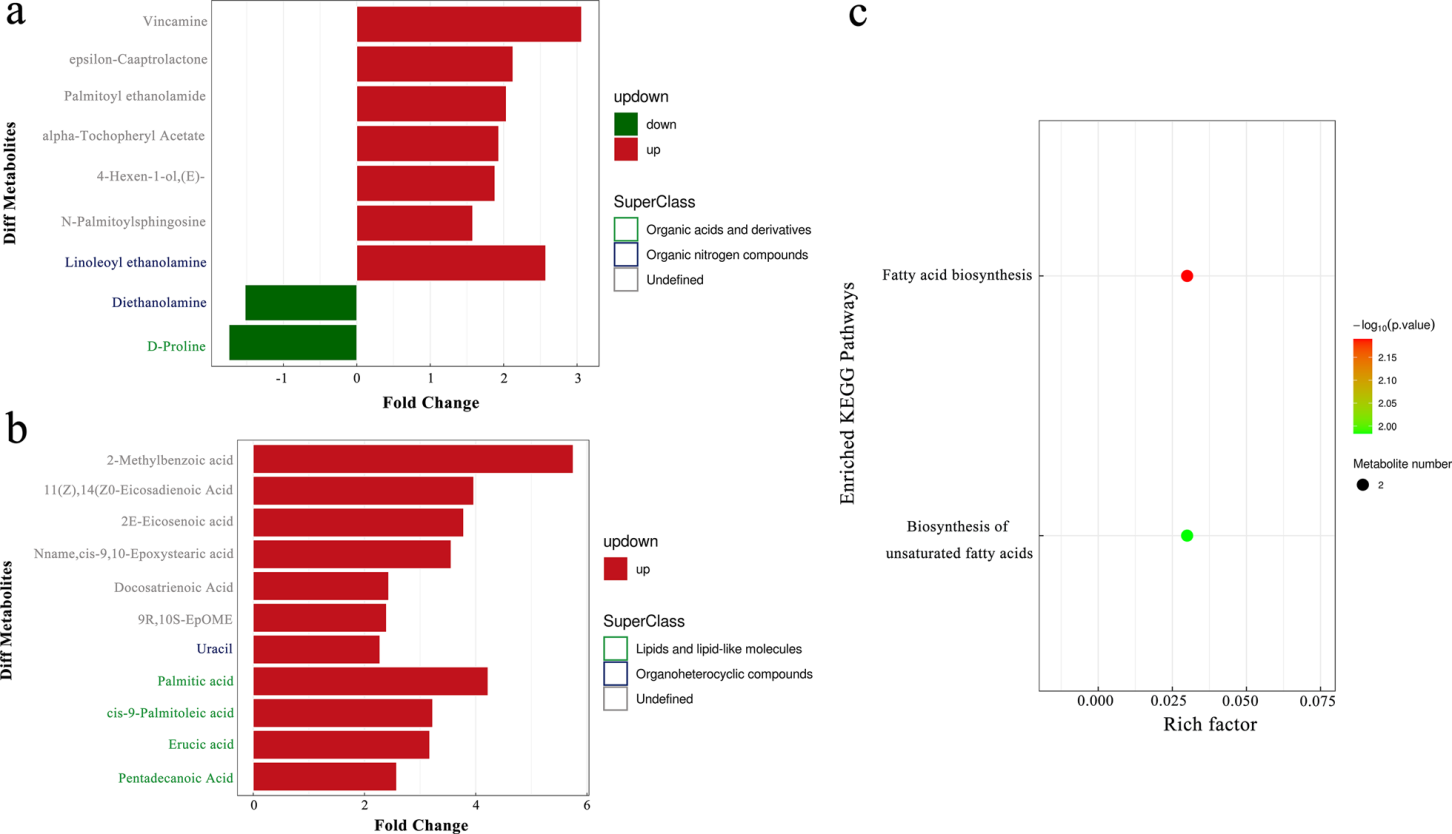
Effect of *E. intestinalis* infection on metabolites and metabolic pathways. **a**, **b** Fold change in the 20 significantly altered metabolites in ESI+ and ESI- modes, respectively. Red and green indicate upregulation (FC > 1.5, *P* < 0.05) and downregulation (FC < 0.67, *P* < 0.05), respectively. **c** The significantly altered metabolic pathways

A total of 269 metabolites were identified by UPLC-Q-TOF/MS in the positive and negative ion modes. Lipids and lipid-like molecules (13.755%) and organic acids and derivatives (13.755%) were the most abundant metabolites. The *E. intestinalis* infection group and control group could be clearly separated into distinct clusters (Fig. 8a), as shown in the PLS-DA plots (*R*^2^*Y* = 0.995, *Q*^2^ = 0.765). According to the permutation test results, the PLS-DA model was proved to have excellent stability, and there was no over-fitting phenomenon (Fig. 8b). Twenty significantly changed metabolites (VIP > 1.0, *P* < 0.05) were successfully identified (Table 1). Metabolites in the same cluster may have similar functions or participate in the same metabolic process (Fig. 8c, d), as shown in the heatmap. Only diethanolamine and D-proline decreased in the *E. intestinalis* infection group (FC < 0.67), while the other 18 metabolites increased (FC > 1.5) (Fig. 9a, b). Among 12 metabolic pathways identified using the KEGG database, fatty acid biosynthesis and biosynthesis of unsaturated fatty acids were significantly enriched in the *E. intestinalis* infection group (*P* < 0.05) (Fig. 9c). Palmitic acid, cis-9-palmitoleic acid, and erucic acid were involved in these two pathways.

**Table 1:** Detailed information on the significantly different metabolites with VIP > 1 and *P* < 0.05

ID	adduct	Name	VIP	Fold change	*P*-value
M300T36	(M+H)+	Palmitoylethanolamide	4.242226378	2.035874826	0.00230537
M539T34_1	(M+H)+	*N*-Palmitoylsphingosine	1.819980103	1.578176379	0.006136909
M70T328	(M+H-2H2O)+	Diethanolamine	1.695962665	0.657442526	0.006342091
M355T408	(M+H)+	Vincamine	2.192763661	3.060522423	0.011041246
M239T361	(2M+K)+	4-Hexen-1-ol, (E)-	2.271122144	1.880983725	0.012781622
M116T324	(M+H)+	D-Proline	2.435333595	0.574689467	0.014247303
M114T347	M+	Epsilon-captrolactone	1.380551211	2.126749801	0.025344005
M324T35	(M+H)+	Linoleoyl ethanolamide	2.57189053	2.572377334	0.0352202
M490T31	(M+NH4)+	Alpha-tochopheryl acetate	3.928309051	1.932374925	0.041368566
M307T39_2	(M-H)-	11(Z),14(Z)-Eicosadienoic acid	1.774253686	3.963155204	0.000655281
M135T131	(M-H)-	2-Methylbenzoic acid	1.148096399	5.750950297	0.005014043
M309T39	(M-H)-	2E-Eicosenoic acid	2.549886786	3.781813861	0.007518555
M253T43	(M-H)-	cis-9-Palmitoleic acid	3.457076984	3.224863289	0.007814621
M241T44	(M-H)-	Pentadecanoic acid	5.545499235	2.576545715	0.007838743
M337T39	(M-H)-	Erucic acid	1.835970829	3.172043697	0.008576675
M333T39	(M-H)-	Docosatrienoic acid	1.224860181	2.433629705	0.01186125
M511T42	(2M-H)-	Palmitic acid	1.486694566	4.220094839	0.01371454
M297T48	(M-H)-	Nname,cis-9,10-Epoxystearic acid	5.3933711	3.55597062	0.013789698
M111T86	(M-H)-	Uracil	2.405671532	2.276246052	0.024974897
M295T53	(M-H)-	9R,10S-EpOME	2.995541502	2.394194928	0.036144385

### Associations between bacterial genera and metabolites

**Fig. 10:**
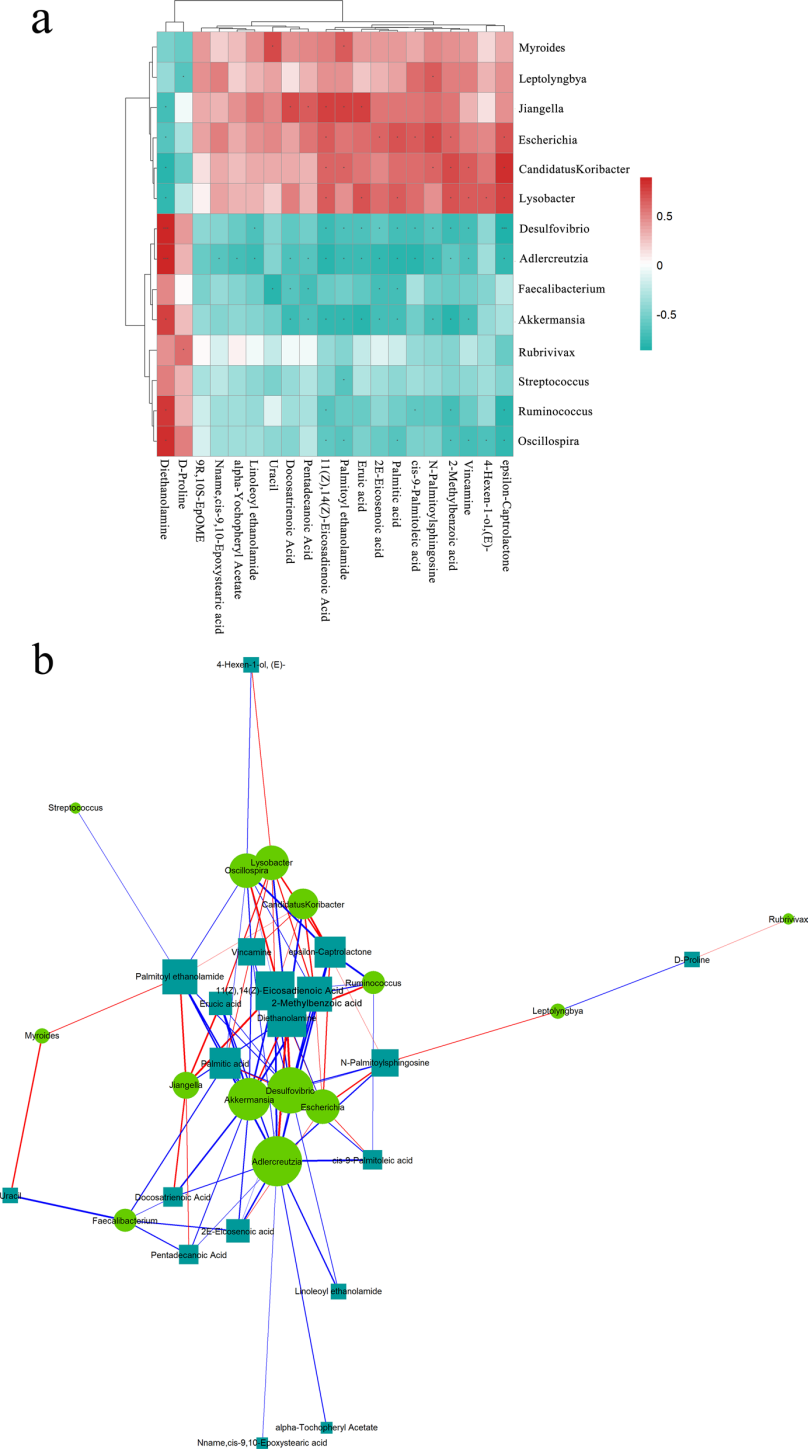
Associations between the significantly altered genera and metabolites. **a** The hierarchical cluster heatmap of genera and metabolites. **b** Correlation network analysis. The circles and rectangles represent significantly changed genera and metabolites, respectively. Red and green indicate positive and negative correlations, respectively. *P < 0.05, **P < 0.01, and ***P < 0.001

Spearman correlation analysis was used to determine the co-variation between the 14 significantly different genera (LEfSe LDA > 2, *P* < 0.05) and the 20 metabolites (VIP > 1, *P* < 0.05). A total of 34 genus–metabolite pairs showed a significantly positive correlation, but 58 pairs had a significantly negative correlation. The correlation for 34 of these pairs was extremely significant (*P* < 0.01). The heatmap showed that *Candidatus Koribacter*, *Lysobacter*, *Escherichia*, *Myroides*, *Jiangella*, and *Leptolyngbya* were negatively correlated with diethanolamine and D-proline, and positively correlated with the other 18 metabolites. The other 8 genera had the opposite correlations with the metabolites. Therefore, the correlations of diethanolamine and D-proline with the gut microbiota were opposite those of the other 18 metabolites (Fig. 10a). Spearman correlation network analysis was performed to further screen the significantly altered metabolites and genera located at key node positions in the network (rho ≥ 0.5, *P* < 0.05). The network diagram showed that *Adlercreutzia*, *Desulfovibrio*, *Escherichia*, and *Akkermansia* were comparatively important genera. Compared with other metabolites, 11(Z), 14(Z)-eicosadienoic acid, 2-methylbenzoic acid, diethanolamine, and palmitoylethanolamide were more strongly correlated with 14 genera (Fig. 10b). In summary, there were strong correlations between the gut microbiota and metabolites. Our findings indicated that significant changes in the species and abundances of gut microbes were caused first, which in turn led to dramatic shifts in metabolites in the rabbits.

## Discussion

*Eimeria intestinalis* mainly parasitizes the jejunum and ileum [2]. It was found that sporozoites first entered the epithelial cells of the duodenum, along the whole length of the small intestine, and subsequently migrated to the crypt epithelium of the ileum [20]. In this study, the whole jejunum was parasitized by *E. intestinalis* in a range of life cycle stages (trophozoite, schizont, microgamont, macrogamont, and oocyst), and the structure of the jejunum was completely destroyed, with many sites of bleeding. Goblet cells are abundant constituents of the epithelium in the intestines and play an important role in mucosal protection [21]. In our results, *E. intestinalis* infection caused extensive loss of goblet cells and reduced glycoprotein secretion. Dkhil [7] also found that there was a significant reduction in goblet cells at the site of *E. papillata* infection in the jejunum. TNF-α is the main mediator of the inflammatory response and can regulate both immune and inflammatory responses [22]. The present results are consistent with those of previous studies [7], which showed significantly increased expression of TNF-α in the jejunum of rabbits infected with *E. intestinalis*. This indicates that the immune cells and epithelial cells in the intestine produce copious amounts of cytokines (i.e., TNF-α) to trigger inflammatory responses after *E. intestinalis* infection.

The jejunum is the longest part of the small intestine and has the largest surface area. As the main site for the digestion and absorption of nutrients, the jejunum is of great importance for maintaining normal physiological functions [23]. In this study, the diversity of the jejunal microbiota decreased because of *E. intestinalis* infection. Firmicutes and Actinobacteria were the dominant phyla in the jejunum of healthy rabbits, consistent with previous reports [24]. In the *E. intestinalis*-infected rabbits, the abundance of Proteobacteria significantly increased compared with that in the control group (from 4.7% to 78.7%), while the abundance of Firmicutes and Actinobacteria decreased. The abundance of Proteobacteria is often found to increase in several intestinal and extraintestinal diseases, most of which exhibit an inflammatory phenotype, and this increase is regarded as a possible microbial signature of disease [25]. The abundance of *Escherichia*, belonging to the Proteobacteria, significantly increased. Previous studies have shown that the abundances of opportunistic pathogenic bacteria, such as *Escherichia*, increased significantly post-*Eimeria* infection [13, 26, 27]. *E. intestinalis* might increase the possibility of subsequent infection by other pathogens and exacerbate jejunal injury, resulting in changes in the diversity of the jejunal microbiota.

Li [28] found that the abundances of *Ruminococcus* and *Eisenbergiella* decreased but that of *Intestinimonas* increased in rabbits infected with a precocious line of *E. intestinalis* (EIP8). In the present study, the abundances of *Oscillospira*, *Ruminococcus*, *Bacteroides*, *Akkermansia*, *Coprococcus*, and *Sarcina* significantly decreased in rabbits infected with *E. intestinalis*. In inflammatory diseases, the abundance of *Oscillospira* is reduced [29]. *Oscillospira* may be able to secrete the important short-chain fatty acid butyrate using host glycans [30], and some members of the Ruminococcaceae are also butyrate producers [31]. Butyrate reduces the permeability of intestinal epithelial cells, thereby inhibiting intestinal inflammation and bacterial translocation [32]. Butyrate also modulates the function of innate immune cells [33]. Treatment of intestinal macrophages with *n*-butyrate can reduce the production of pro-inflammatory mediators such as NO, IL-6, and IL-12 [34]. Therefore, *Oscillospira* and *Ruminococcus* species may play an important role in resistance to coccidia infection by secreting butyrate.

*Akkermansia muciniphila*, belonging to the phylum Verrucomicrobia [35], colonizes the mucosal layer of the gastrointestinal tract [36]. It is reduced substantially in patients affected by inflammatory bowel disease [37]. A recent study showed that supplementation with *Akkermansia muciniphila* or its outer membrane protein Amuc_1100 can regulate immune responses in the spleen, intestines, and mesenteric lymph nodes and reduce the level of inflammation in the intestines [38]. Thus, *A. muciniphila* is probably associated with a protective or anti-inflammatory role in *E. intestinalis* infection. In general, *E. intestinalis* infection disrupts the intestinal flora in rabbits, increases the abundance of some harmful bacteria, and decreases the abundance of some beneficial bacteria. The abundance changes in the flora may weaken the intestinal barrier and increase the possibility of pathogen invasion.

Fecal metabolomics has been used to assess metabolic phenotype variations associated with gut microbiota perturbations in disease development [39]. In this study, the non-targeted metabolomics results showed widespread effects on metabolic profiles in rabbits with *E. intestinalis* infection. A total of 20 significantly altered metabolites were found to contribute to the variations between infected and uninfected rabbits. The metabolites significantly altered in response to *E. intestinalis* infection were lipids. KEGG pathway analysis showed significant dysregulation of lipid metabolism (biosynthesis of fatty acid and unsaturated fatty acids), accompanied by increases in palmitic acid, cis-9-palmitoleic acid, and erucic acid. Lipids are essential structural components of biological membranes and play important roles in the mechanisms of apoptosis, material exchange, energy transfer, and signal transduction [40, 41]. The damage caused by *E. intestinalis* to the rabbit intestinal tract mainly occurred during the schizogony stage [42], with destruction of intestinal villi after several generations of merozoite production. Therefore, the anomalous changes in lipid metabolites may result from the destruction of intestinal epithelial cell structure by *E. intestinalis* infection, leading to dysfunction of absorption.

All 20 significantly altered metabolites except for diethanolamine and D-proline, the other metabolites increased in the *E. intestinalis* infection group. Proline can promote epithelial cell proliferation, maintain intestinal integrity and improve barrier function after stress injury [43]. Huang [27] also reported that proline significantly decreased in mice infected with *Eimeria falciformis*. The majority of glycerophospholipids were significantly reduced in response to *Schistosoma japonicum* infection [44]. Here, no significant abundance differences in the glycerophospholipid metabolic pathway were found between the two groups, but diethanolamine, which is involved in this pathway, was significantly reduced.

Palmitoylethanolamide can inhibit mitochondrial dysfunction, reduce cytokine production, ameliorate the inflammatory state, and limit oxidative stress [45]. Vincamine can obviously inhibit the production of inflammatory mediators and oxidative stress through the activation of thioredoxin reductase [46]. Tocopherol is a hydrolysate of vitamin E that is capable of modulating the gut microbiota in mice with DSS-induced colitis and mitigating intestinal inflammation [47]. Pretreatment with oleoylethanolamide was found to reduce intestinal inflammation and innate/adaptive immune responses affected by alcohol binges [48]. The increased levels of these metabolites are related to the immunoprotection stimulated by *E. intestinalis* infection. Moreover, pathogenic bacteria in the gut can produce uracil, which activates host dual oxidase to produce antimicrobial reactive oxygen species [49]. However, Ng Hublin [50] reported that the level of the pyrimidine uracil was significantly lower in mice infected with *Cryptosporidium parvum*.

The above analysis indicates that the alterations in the gut microbiota and metabolites in *E. intestinalis*-infected rabbits occurred in the positive direction. *Adlercreutzia*, *Akkermansia*, *Desulfovibrio*, *Escherichia*, *Oscillospira*, and *Lysobacter* were the important genera located at key nodes in the network. For example, vitamin E (a-tocopherol) consumption affects the Proteobacteria and Verrucomicrobia composition, corresponding to that of *Escherichia coli* and *Akkermansia muciniphila* at the species level [51]. In conclusion, *E. intestinalis* infection increased intestinal inflammation and destroyed intestinal homeostasis at the parasitized sites, causing significant changes in the gut microbiota, which in turn led to corresponding changes in metabolites.

## Conclusions

In the present study, jejunal epithelial cells were parasitized by a large number of *E. intestinalis* oocysts and other life cycle stages. *E. intestinalis* infection destroyed the jejunal structure, caused extensive loss of goblet cells, reduced glycoprotein secretion, and increased the expression of TNF-α. It was demonstrated that both the gut microbiota and metabolites were significantly altered in rabbits infected with *E. intestinalis*. *Eimeria intestinalis* infection reduced the diversity of the jejunal microbiota and was related to 14 different jejunal microbiota at the genus level. Metabolomics analysis showed that *E. intestinalis* infection altered 20 metabolites and two metabolic pathways, with lipid metabolism as the major disrupted metabolic pathway. In addition, Spearman correlation analysis revealed that 92 genus–metabolites pairs had a significant correlation. In conclusion, *E. intestinalis* infection damaged the jejunal structure, increased intestinal inflammation, and perturbed the gut microbiota and metabolic homeostasis. The altered microbiota and fecal metabolites may become potential biomarkers and provide a reference for the diagnosis of coccidiosis infection.

## Data Availability

The 16S rRNA sequencing data in this study were submitted to the NCBI Sequence Read Archive (SRA) under BioProject PRJNA 818494.
